# The increased expression of fatty acid-binding protein 9 in prostate cancer and its prognostic significance

**DOI:** 10.18632/oncotarget.12635

**Published:** 2016-10-13

**Authors:** Majed Saad Al Fayi, Xiaojun Gou, Shiva S. Forootan, Waseem Al-Jameel, Zhengzheng Bao, Philip R. Rudland, Philip A. Cornford, Syed A. Hussain, Youqiang Ke

**Affiliations:** ^1^ Molecular Pathology Laboratory, Department of Molecular and Clinical Cancer Medicine, Liverpool University, the Cancer Research Centre Building, Liverpool, United Kingdom; ^2^ Sichuan Antibiotics Industrial Institute, Chengdu University, Chengdu, China; ^3^ Department of Medical Laboratory Sciences, College of Applied Medical Sciences, King Khalid University, Abha, Kingdom of Saudi Arabia; ^4^ Department of Biochemistry, Liverpool University, Liverpool, United Kingdom

**Keywords:** prostate cancer, FABP9, gleason scores, prognosis, PSA

## Abstract

In contrast to numerous studies conducted to investigate the crucial role of fatty acid binding protein 5 (FABP5) in prostate cancer, investigations on the possible involvement of other FABPs are rare. Here we first measured the mRNA levels of 10 FABPs in benign and malignant prostate cell lines and identified the differentially expressed *FABP6* and *FABP9* mRNAs whose levels in all malignant cell lines were higher than those in the benign cells. Thereafter we assessed the expression status of FABP6 and FABP9 in both prostate cell lines and in human tissues. FABP6 protein was overexpressed only in 1 of the 5 malignant cell lines and its immunostaining intensities were not significantly different between benign and malignant prostate tissues. In contrast, FABP9 protein was highly expressed in highly malignant cell lines PC-3 and PC3-M, but its level in the benign PNT-2 and other malignant cell lines was not detectable. When analysed in an archival set of human prostate tissues, immunohistochemical staining intensity for FABP9 was significantly higher in carcinomas than in benign cases and the increase in FABP9 was significantly correlated with reduced patient survival times. Moreover, the increased level of staining for FABP9 was significantly associated with the increased joint Gleason scores (GS) and androgen receptor index (AR). Suppression of FABP9 expression in highly malignant PC3-M cells inhibited their invasive potential. Our results suggest that FABP9 is a valuable prognostic marker to predict the outcomes of prostate cancer patients, perhaps by playing an important role in prostate cancer cell invasion.

## INTRODUCTION

Prostate cancer is the most common male cancer in the developed countries and a worldwide male health threat [1, 2]. The incidence and mortality rate of prostate cancer increased throughout the entire 1980s and peaked in 1991 [3]. Since 1992, the serum prostate-specific-antigen (PSA) screen was used on a wide population use of this marker helped in early diagnosis of prostate cancer and thus contributed to improvements in subsequent 5-year survival rates [4–6]. Currently PSA is the most common marker used for both diagnosis and treatment management [7]. Although the widespread screening for PSA did lead to a degree of decline in mortality rates in some countries, many prostate cancer cases were not picked up by PSA screening in other countries. Several separate studies even suggested that PSA screening had only very limited or even no survival benefits [5, 8, 9], when a PSA cutoff level of 4 ng/ml was used to recommend a prostate biopsy (or not). Since PSA is produced and secreted by both benign and malignant prostate cells, the serum level of PSA can only reflect the size of the prostate gland, it cannot be used to distinguish the benign or malignant nature of the cells. Thus PSA levels may also be increased in some benign prostatic diseases, such as prostatitis and benign prostate hyperplasia (BPH). Thus the real benefit of the widespread use of PSA screening is still debatable and the reliability of PSA as a prostate cancer marker is in serious question [5, 8, 9]. Therefore, a reliable and accurate diagnostic or prognostic marker [6, 10, 11] that can be used to distinguish between benign and malignant prostatic disease is urgently needed for making speedy and correct treatment decisions [5, 12, 13].

To investigate the molecular mechanisms involved in the malignant progression of prostate cancer cells and to identify possible novel markers, we characterised the role of FABP5 and demonstrated its crucial function in promoting tumorigenicity and metastasis of prostate cancer [10, 14]. After FABP5 was proven to be a prognostic marker and potential treatment target [15–18], a frequently asked question was whether other FABP family proteins were also diagnostic or prognostic markers for prostate cancer. Twelve members of the FABP family with sizes between 14 to 15 kDa have been identified so far and 2 of them are restricted to fish. The other 10 FABPs are expressed in different human organs either singly or co-expressed with 1 or more other family members [19–21]. FABPs are intracellular lipid-binding proteins that bind intracellular hydrophobic ligands such as long-chain fatty acids. FABPs have been suggested to be involved in secretion, uptake and intracellular transport of lipids to subcellular-organelles [19, 20]. FABPs have also been suggested to control cancer growth through co-ordination with other fatty acid transporters and carcinogens [22], steroids [23], hormones [24] and their derivatives [21].

In this study, we have investigated whether FABPs other than FABP5 can be used as diagnostic or prognostic markers; these may be involved in initial development and in malignant dissemination of prostate cancer cells. We first measured mRNA levels of 10 FABPs in benign and malignant human prostate epithelial cell lines with quantitative RT-PCR and found that *FABP6* and *FABP9* mRNA levels in all malignant cell lines tested were higher than those in the benign cell lines. We then used Western blot and immunohistochemical staining to assess the expression status of FABP6 and FABP9 proteins in both prostate cell lines in culture and human prostatic tissues. The differences in expression profiles between benign and malignant cell lines and prostatic tissues were fully assessed and their possible value in diagnosis and prognosis have been systematically analysed.

## RESULTS

### Relative levels of mRNAs of different *FABPs* in prostate cells

**Figure 1:**
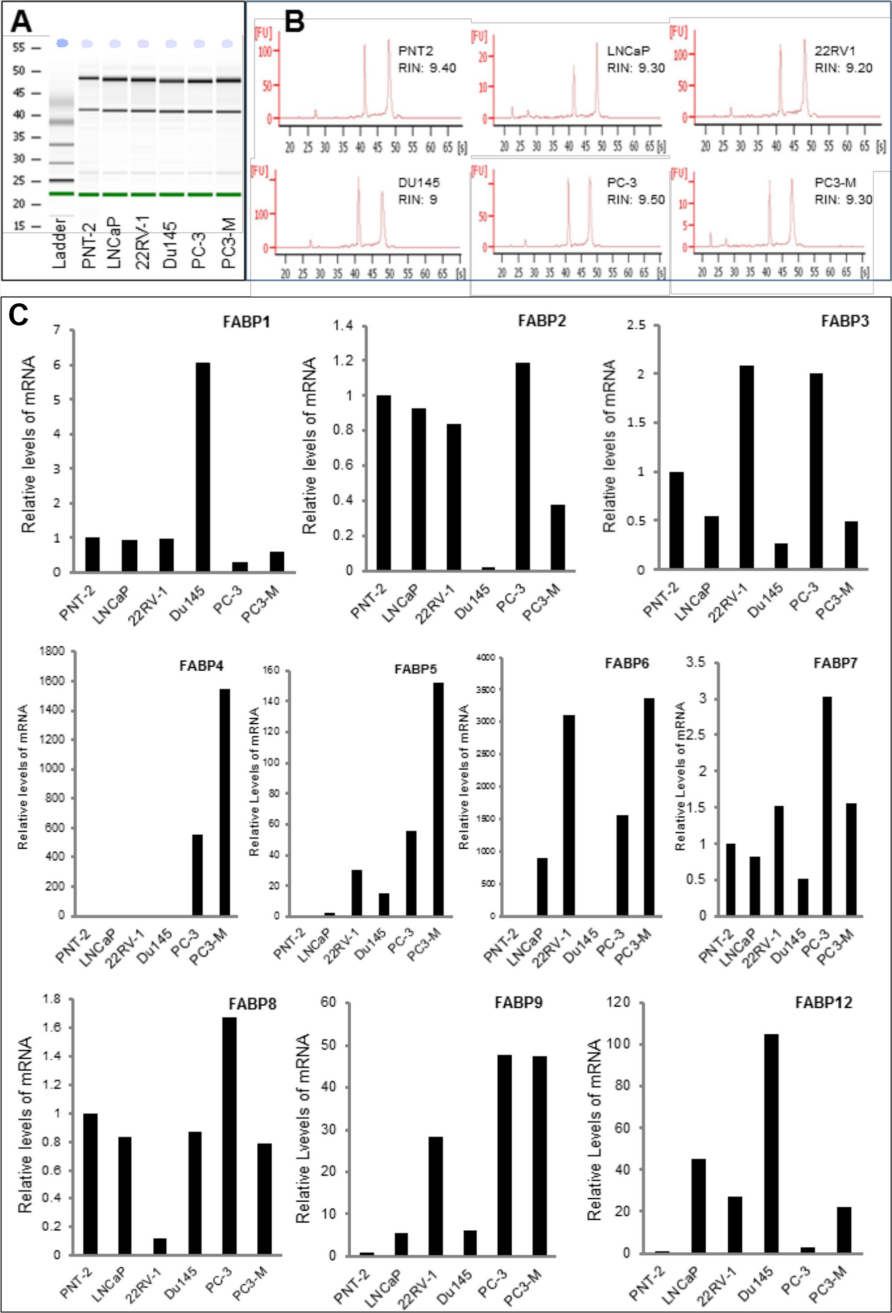
Quantitative PCR analysis of levels of FABP mRNAs in benign and malignant prostate epithelial cells Benign cell line PNT-2, weakly malignant cell line LNCaP, moderately malignant cell line 22RV-1 and highly malignant cell lines Du145, PC-3 and PC3-M were cultured to 80% confluence and harvested for mRNA extraction. The quality of the mRNA was assessed by a 2100 Bio-analyser. **A.** the electrophernograms of the gel-like images of mRNAs from 6 cell lines and from the RNA ladder (marker). **B.** the double peaks representing 18S and 28S sub-units and the RNA integrity numbers (RIN) of the samples from different cell lines. **C.** relative levels of FABP mRNAs detected in benign and malignant prostate cell lines.

Messenger RNAs of 10 different FABPs were isolated; their levels in the benign PNT-2 cells and 5 malignant prostate cell lines were measured by quantitative RT-PCR and the results are shown in Figure 1. The high quality of mRNA from each cell line was confirmed by two clear bands representing both 18S and 28S sub rRNA units (Figure 1A). The high RNA integrity number (RIN) of the samples (between 9.0 and 9.6) showed that the total RNAs were intact (Figure 1B). The relative levels of mRNAs of 10 different FABPs in 6 different cell lines are shown in Figure 1C. Assessing transcription profiles between the benign and malignant cell lines showed that *FABP4, FABP5, FABP6, FABP9* and *FABP12* exhibited clearly higher levels in all malignant cell lines. For *FABP1, FABP3, FABP2 FABP7* and *FABP8,* no clear and unified differences in their mRNA levels were identified when malignant were compared with benign cell lines.

### Expression of FABP6 and FABP9 in prostate cells at the protein level

**Figure 2:**
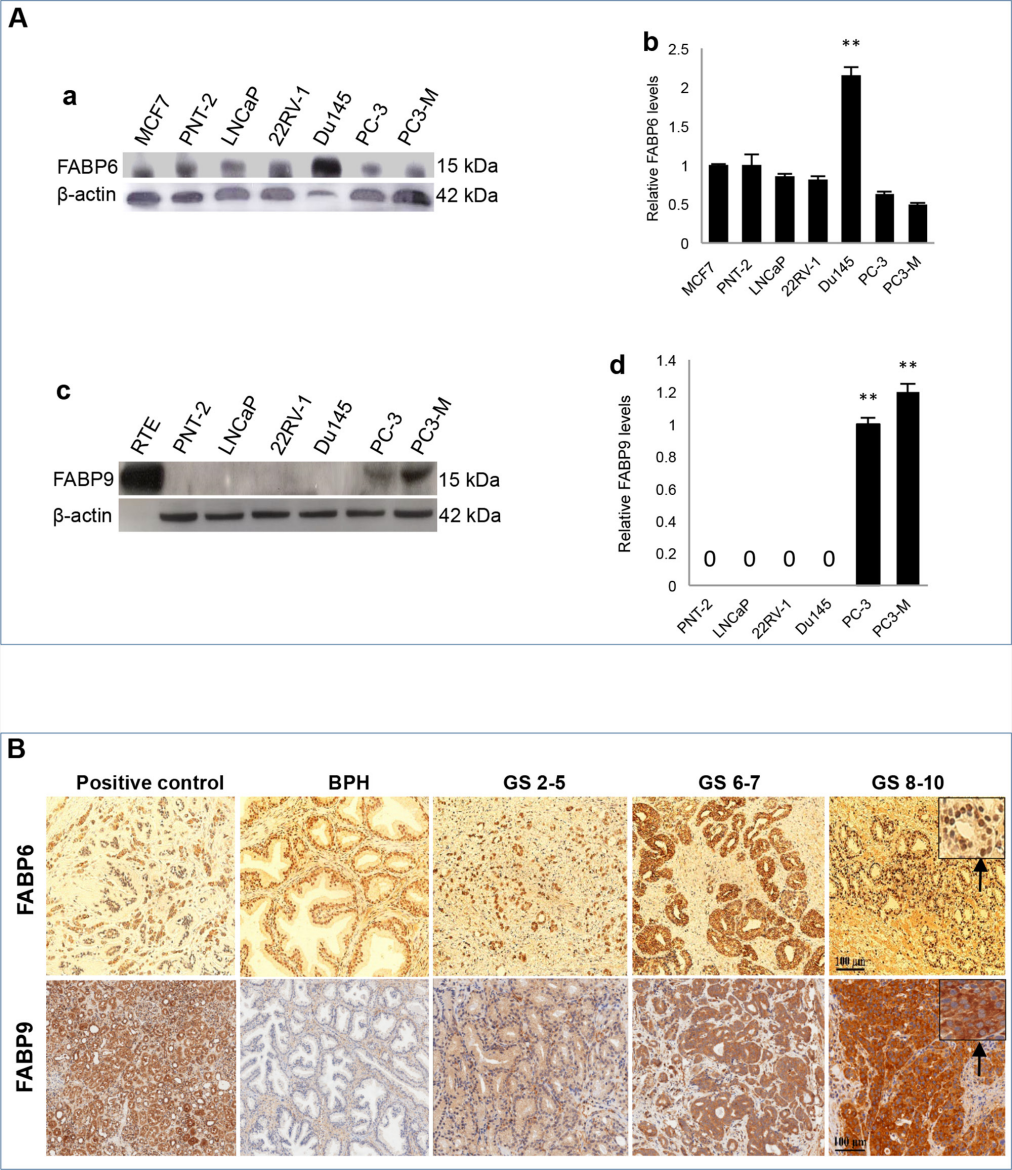
Expression of FABP6 and FABP9 in prostate cell lines and tissues **A.** Measurement of FABPs in benign and malignant prostate cells. **a.** Western blot analysis of FABP6 in benign and malignant prostate cells. **b.** relative levels of FABP6 in different cell lines. The level of FABP6 in the benign PNT-2 cells was set at 1.0; levels in other cell lines were obtained by relating to that in PNT-2 cells. The positive control (MCF-7) was suggested by the manufacturer of the antibody. The results were obtained from 3 separate experiments (mean ± SD). **c.** Western blot analysis of FABP9 in benign and malignant prostate cells. **d.** relative levels of FABP9 in different cell lines. Since the expression in PNT-2, LNCaP, 22RV-1 and Du145 is not detectable, their levels were expressed as “0”. The level in PC-3 cells was set at 1.0, the level in PC3-M cells was obtained by relating to that in PC-3. The results were obtained from 3 separate experiments (mean ± SD). **B.** Immunohistochemical detection of FABP6 and FABP9 in benign and malignant prostate tissues. Positive controls for FABP6 and FABP9 were breast cancer tissue and normal kidney tissue respectively (x100). For anti-FABP6, only nuclear staining was observed (identified in the insert by the arrow). For anti-FABP9, only cytoplasmic staining was observed (identified in the insert by the arrow). Carcinomas were divided into three categories according to their combined GS: weakly malignant (or GS ≤5), moderately (or GS 6-7) and high malignant (or GS 8-10) tissues.

Differential expression of FABPs in levels of protein is shown in Figure 2. When analysed by Western blot (Figure 2A), a single 14 kDa FABP6 band was detected in all prostate cell lines as well as a positive control MCF7 breast cancer cells (Figure 2A: a). Quantitative analysis showed that the level of FABP6 in highly malignant Du145 cells was more than 2 times of that in the benign PNT-2 cells (Student's t-test, *p* = 0.01). The levels of FABP6 in low malignant LNCaP cells, moderately malignant 22RV-1 cells, highly malignant PC-3 and PC3-M cells were lower than that in PNT-2 cells (Figure 2A: b), but the differences were not significant (Student's t-test, *p*>0.05). FABP9 protein running as a single band at 14 kDa was highly expressed in 2 of the 5 malignant PC-3 and PC3-M cells, but its expression in other prostate cells including benign PNT-2 cells was not detectable (Figure 2A: c). Further quantitative analysis showed (Figure 2A: d) that the level of FABP9 in the highly malignant cells PC3-M was a significant 20% higher than that in PC-3 (Student's t-test, *p*= 0.006).

### Detection of FABP6 and FABP9 in prostate tissues

The expression of FABP6 and FABP9 in the control, BPH and carcinoma tissues was detected by immunohistochemical (IHC) staining and representative staining patterns are shown in Figure 2B and quantified in Table 1. IHC showed nuclear staining only on BPH and carcinoma cells with antibodies to FABP6 (identified by the arrow), no cytoplasmic staining was observed (Figure 2B). Results of anti-FABP6 staining were shown in Table 1A. Nuclear staining was observed in all 33 BPH tissues (100%): 3 (9%) stained weakly, 25 (76%) stained moderately, and 5 (15%) stained strongly. Among the 92 adenocarcinomas, 28 (30%) were unstained, 11 (12%) stained weakly, 23 (25%) stained moderately and 30 (33%) stained strongly. Although FABP6 was detected in the nucleus of the cells, the staining intensities between BPH and carcinoma tissues were not significantly different (χ^2^ test, *p*>0.08).

Table 1Expression status of FABP6 and FABP9 in benign and malignant prostate tissues

**Table 1A:** Immunohistochemical stains of tissues with antibody against FABP6

FABP6	Nuclear stain intensities ^a^				No. of cases
Tissues	0	+	++	+++	
BPH	0	3	25	5	33^b^
Carcinomas (Total)	28	11	23	30	92^b^
GS^c^ ≤5	14	0	6	3	23
GS^c^ 6-7	10	4	11	10	35
GS^c^ 8-10	4	5	8	17	34

a Staining was observed only in the nucleus of the cells.

b Total BPH were 36 and total carcinomas were 97, but some cases were excluded because of technical reasons.

c Combined GS.

**Table 1B:** Immunohistochemical stains of tissues with antibody against FABP9

FABP9	Cytoplasmic stain intensities ^a^				No. of cases
Tissues	0	+	++	+++	
BPH	25	11	0	0	36
Carcinomas (Total)	0	31	39	16	86^b^
GS^c^ ≤5	0	22	6	0	28
GS^c^ 6-7	0	7	13	5	25
GS^c^ 8-10	0	2	20	11	33

a Staining was observed only in the cytoplasm of the cells.

b Total carcinomas were 97, but some cases were excluded because of technical reasons.

c Combined GS.

The staining of FABP9 in carcinoma tissues was cytoplasmic only (identified by the arrow) (Figure 2B) and the results are shown in Table 1B. Among 36 BPH cases 25 (70%) were unstained and 11 (30%) stained weakly. Among 86 adenocarcinomas, staining was weak in 31 (36%), moderate in 39 (46%) and strong in 15 (18%) cases (Table 1B). No nuclear staining was observed in any BPH and carcinoma samples. Comparing the FABP9 expression between BPH and carcinomas, the staining intensity in carcinoma tissues was significantly stronger (χ^2^ test, *p*<0.0001).

### Correlation of FABP9 expression to GS

According to their GS, carcinomas were divided into 3 groups: low (GS ≤5), moderate (GS 6-7) and high malignant (GS 8-10), as shown in Table 1B. Of the 28 weakly malignant carcinoma tissues, 22 (78%) stained weakly, 6 (22%) stained moderately, none of them stained strongly. Among the 25 moderately malignant cases with GS 6-7, 7 (27%) stained weakly, 13 (53%) stained moderately and 5 (19%) stained strongly. Of the 33 highly malignant cases with GS 8-10, 2 (6%) stained weakly, 20 (63%) stained moderately and 11 (31%) stained strongly. The intensity of FABP9 staining in moderately malignant cases was significantly higher than that in low malignant cases (χ^2^ test, *p*<0.00001). Highly significant difference was also observed in staining intensities between low malignant cases and highly malignant cases (χ^2^ test, P<0.00001). When the staining intensities between the moderately malignant and the highly malignant cases were compared, the difference was not statistically significant (χ^2^ test, *p* =0.09).

### Relationship between patient survival and levels of FABP9, GS, AR index and PSA

**Figure 3:**
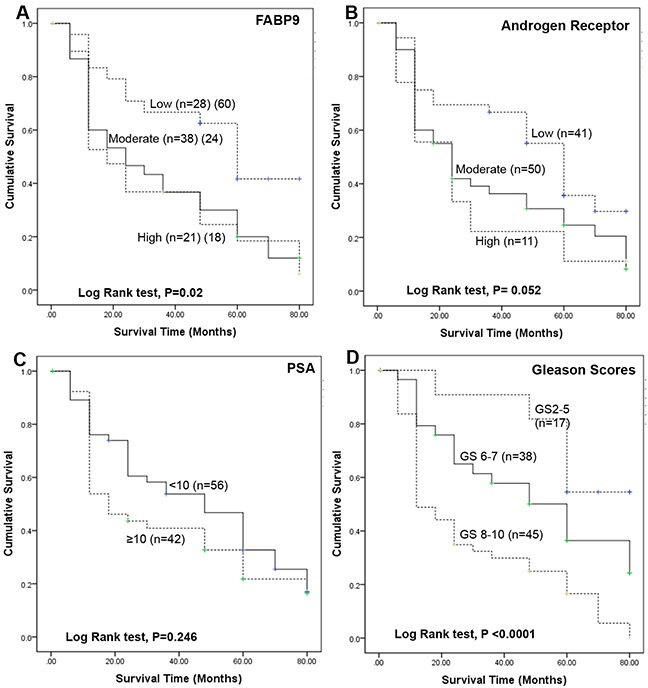
Kaplan-Meier survival curves of patients with prostate cancer The cumulative survival of patients was plotted against the time in months for different levels of 4 parameters. **A.** different GS: group 1, GS ≤5 (n=17); group 2, GS 6 to 7 (n=38); group 3, GS 8 to 10 (n=45). **B.** different AR indices: low group, <4 (n=41); moderate group, 4-6 (n=50); high group, 6-9 (n=11). **C.** different levels of PSA: group 1, PSA <10 ng/ml (n=38) and group 2, PSA ≥10 ng/ml (n=64). **D.** different levels of staining for FABP9: group 1, weakly positive (n=28); group 2, moderately positive (n=38); group 3, strongly positive (n=21).

The cumulative survival of patients was plotted against the time in months using Kaplan-Meier survival graphs for different levels of the 4 parameters and the significance for each parameter was assessed by log-rank tests as shown in Figure 3. When the correlation between patient survival time and the FABP9 staining intensity was assessed (Figure 3A), the median survival time for patients with weak staining for FABP9 was 60 months which was significantly longer than those patients with moderate (24 months) and strong (18 months) staining for FABP9, respectively. Overall, the increased FABP9 staining intensity was significantly correlated with reduced patient survival time (log-rank test, *p* = 0.02).

When the correlation between the patient survival time and AR index level was assessed (Figure 3B), the median survival time for patients with weak, moderate and strong staining for FABP9 was 60, 24 and 24 months, respectively. The overall increase in intensity of AR staining was of borderline significance in correlation with reduced patient survival time (log-rank test, *p* = 0.052).

When the correlation between patient survival time and the blood level of PSA was assessed (Figure 3C), the median survival time for patients with low (<10ng/ml) and high (≥10ng/ml) PSA levels were 48 and 18 months, respectively. The difference was not statistically significant (log-rank test, *p* = 0.246).

When the relationship between patient survival time and GS was assessed (Figure 3D), the median survival times for patients with low GS (80 months) was significantly longer than for those patients with moderate (60 months) and high GS (12 months), respectively. The increased GS was significantly correlated with reduced patients' survival time (log-rank test, *p* < 0.0001).

### Correlation of FABP9 expression with levels of GS, AR index and PSA

**Figure 4:**
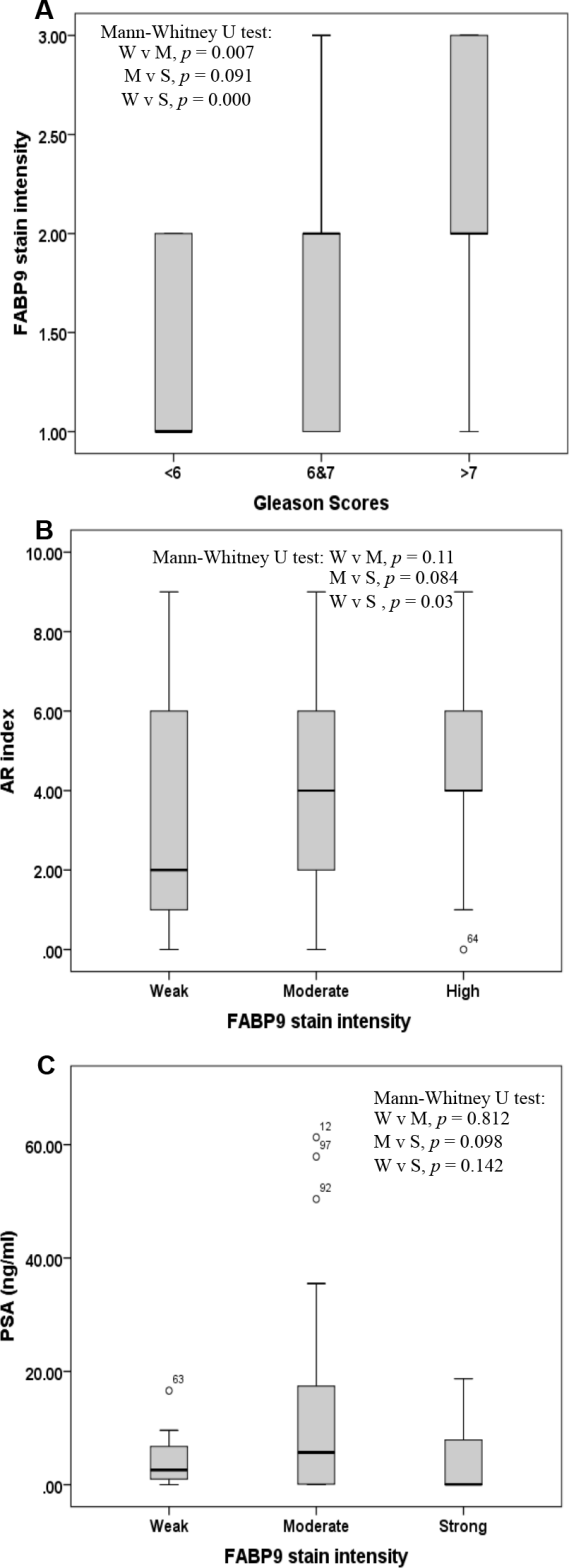
Box plot of correlations amongst 3 different variables **A.** Correlation between the stain intensity for FABP9 and the Gleason score (GS) in 3 groups of patients with prostate cancer: GS < 6 (low), GS 6 to 7 (moderate) and GS 8 to 10 (high). **B.** Correlation between stain intensity for FABP9 and androgen receptor (AR) index in 3 groups of patients with prostate cancer: weak (W), moderate (M) and high (S) levels of FABP9. **C.** Correlation between stain intensity for FABP9 and prostatic specific antigen (PSA) level in 3 groups of patients with prostate cancer: weak (W), moderate (M) and high (S) levels of FABP9.

Assessment of correlation between the intensity of staining for FABP9 and GS, AR index and PSA level is shown in Figure 4. Correlation between the staining intensity of FABP9 and GS is illustrated by box plot (Figure 4A) and the result assessment showed that expression of FABP9 was significantly higher in cases with high GS than in those cases with low GS (Mann-Whitney *U* test, *p* < 0.001). The staining intensity for FABP9 in cases with moderate GS (6 to 7) was significantly higher than that in cases with low GS (Mann-Whitney *U* test, *p* = 0.007). However, the difference in staining for FABP9 between cases with high GS and those with moderate GS was not significantly different (Mann-Whitney *U* test, *p* = 0.091).

Box plot of the correlation between staining intensity for FABP9 and AR index showed that the AR index level was significantly higher in cases with strong staining for FABP9 than in those cases with weak staining for FABP9 (Figure 4B) (Mann-Whitney *U* test, *p* = 0.03). However, the differences in AR index levels either between cases with moderate and weak FABP9 staining (Mann-Whitney *U* test, *p* = 0.11) or between cases with strong and weak staining for FABP9 (Mann-Whitney *U* test, *p* = 0.084) were not statistically significant.

For the correlation between the staining intensity of FABP9 and PSA level (Figure 4C), box plots showed that the differences in PSA levels between cases with strong and weak stains for FABP9 (Mann-Whitney *U* test, *p* = 0.142), between strong and moderate stains (Mann-Whitney *U* test, *p* = 0.098) and between moderate and weak stains for FABP9 (Mann-Whitney *U* test, *p* = 0.812) were not significantly different.

### FABP9 knockdown and its effect on invasiveness

**Figure 5:**
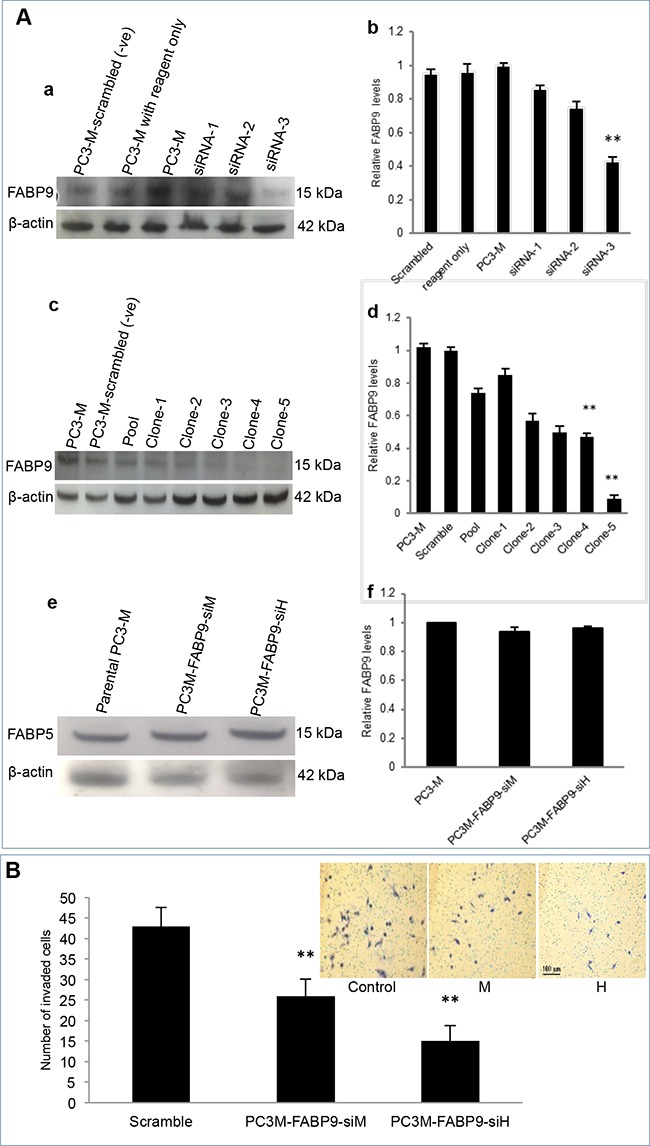
Knockdown of FABP9 mRNA in highly malignant prostate cancer cells and testing the invasiveness of the transfectants expressing reduced levels of FABP9 **A.** Effect of mRNA knockdown on FABP9 expression in PC3-M cells. **a.** Western blot analysis of FABP9 expressed in PC3-M cells and cells transiently transfected with 3 different siRNAs. To standardize the immune reactions, β-actin antibody was incubated with the same blot. **b.** relative levels of FABP9 in PC3-M cells after transient transfection. The level of FABP9 in parental PC3-M was set at 1.0 and levels expressed in other transiently-transfected cells with the controls and different siRNAs were obtained by relating to that in parental cells. **c.** Western blot analysis of FABP9 in control PC3-M cells and 5 different clones generated by transfection with the shRNA based on siRNA-3. To standardize the immune reactions, β-actin antibody was incubated with the same blot. **d.** The relative levels of FABP9 in PC3-M transfected with scramble shRNA control was set at 1.0 and levels expressed in other transfected cell lines were obtained by comparing with the control. **e.** Western blot analysis of FABP5 in transfectants expressing reduced levels of FABP9. **f.** The relative levels of FABP5 in PC3-M cells and transfectants expressing reduced levels of FABP9. **B.** The effect of FABP9 suppression on invasiveness of transfectant cells. The results (mean ± SD) were obtained from 3 separate experiments. Insert picture: 3 panels represent the invasiveness of the control, moderately suppressed (M) and highly suppressed (H) cells respectively.

Knockdown of FABP9 mRNA in highly malignant prostate cancer cells and testing the invasiveness of the transfectants expressing reduced levels of FABP9 (not via FABP5 suppression) are shown in Figure 5. Western blot analysis was used to assess the effect of mRNA knockdown on FABP9 expression in PC3-M cells (Figure 5A). When the PC3-M cell line was transiently transfected for 48 hours with 3 different siRNAs, Western blots showed that the levels of FABP9 expression after transfection were reduced (Figure 5A. a). Quantitative analysis showed that the levels of FABP9 in scramble control and PC3-M treated with transfection reagent alone were similar to that in the parental PC3-M cells. The relative levels in the other three PC3-M transfectants with siRNA 1, 2 and 3 were (means±SD) 0.86±0.14, 0.75±0.35 and 0.45±0.30, respectively. The most efficient suppression was achieved by siRNA-3 (up to 60%) (Student's t-test, *p*< 0.0001) (Figure 5A. b). Therefore, siRNA-3 was identified as the most effective suppresser and its sequence was used to design double-stranded shRNA for stable transfection.

For stable transfection, shRNAs were cloned into the psiRNA-h7SKGFPzeo plasmid and then transfected into the PC3-M cell line to knockdown stably *FABP9* mRNA. When Western blots were used to measure the level of FABP9 from different individual colonies of transfectants, a single 14 kDa FABP9 band was detected and levels of FABP9 in scramble control PC3-M, parental PC3-M and 5 single transfectant clones are shown in Figure 5A-c. Further quantitative analysis showed that the level of FABP9 in the scramble control was similar to that in the parental PC3-M cells. The relative levels of FABP9 in 1-5 single colonies were reduced by 25%, 15%, 43%, 50% and 91%, respectively (Figure 5A. d). Thus cell lines established from clone 4 and clone 5 were selected as moderately (PC3M-FABP9-siM) and highly (PC3M-FABP9-siH) suppressed transfectant lines. To confirm that the suppression was not via FABP5, Western blot analysis was used to assess the effect of FABP9 mRNA knockdown on FABP5 expression in PC3-M cells (Figure 5A. e). Quantitative analysis showed that the levels of FABP5 in PC3M-FABP9-siM and PC3M-FABP9-siH were similar to that in the parental PC3-M cells (Figure 5A. f). The effect of FABP9-supression on invasiveness of prostate cancer cells was evaluated by an invasion assay, as shown in Figure 5B (inserted pictures). The mean number of invaded cells from scramble control, PC3M-FABP9-siM and PC3M-FABP9-siH were 43±7, 26±4 and 15±5 respectively, representing significant reductions by 39.5% (χ^2^ test, *p* = 0.008) and 65.1% (χ^2^ test, *p* = 0.001), respectively.

## DISCUSSION

FABPs are intracellular lipid-binding proteins that bind intracellular hydrophobic ligands such as saturated or unsaturated medium- or long-chain fatty acids and consist of at least 12 members. Previous studies showed that some FABPs were implicated in progression of several cancer types and could be used as tumour biomarkers [25–27]. FABP5 played a crucial promoting role in tumorigenicity and metastasis of prostate cancer [10, 14]. It was also involved in malignant progression of pancreatic cancer [28]. It was reported that the FABP5 and FABP6 were overexpressed and might play promoting roles in colorectal cancer [27, 29]. FABP1 was shown to play a key role in breast cancer cells and has the potential to serve as a diagnostic marker [30]. FABP7 was shown to be a specific biomarker for renal cell carcinomas [31]. In addition, increased expression of FABP7 was associated with proliferation and invasion of melanoma cells [32]. Although functional characterisation work and other studies demonstrated that FABP5 and other FABPs may be prognostic markers and treatment targets for different cancer types, the potential involvement of other FABPs in malignant progression of prostate cancer had not been fully investigated. Amongst the 12 members of FABP family proteins, FABP10 and FABP11 are restricted to fish only and not expressed in Homo sapiens [33–35]. Thus in this study, we measured mRNA levels of the other 10 different *FABPs* in prostate epithelial cells to assess whether any of them were differentially expressed between benign and malignant phenotypes. The results showed that *FABP4, FABP5, FABP6, FABP9* and *FABP12* exhibited clearly higher levels in all tested malignant cell lines compared to their levels in the benign PNT-2 cells (Figure 1). For the remained of the *FABPs*, no clear differences were observed and therefore they are unlikely to be involved in carcinogenesis of prostate cancer.

Amongst the other differentially expressed *FABPs*, FABP4 was released from adipocytes, implicated in obesity [36] and its possible involvement in the malignant progression of prostate cancer has been investigated previously [37]. Although *FABP6* was differentially expressed at the level of its mRNA, its expression at the protein level was not significantly different between the BPH and the carcinoma tissues (Table 1A) and it was expressed in nucleus. Most FABPs are fatty acid transporters and are localised mainly in cytoplasm (not fixed in a specific site) and move in and out of cell membrane to transport fatty acids. Why FABP6 is localised in nucleus is not known, since it is not differentially expressed between benign and malignant tissues, it is unlikely to be related to prostate cancer. FABP12 is the most recently discovered member of the FABP family, its mRNA level in malignant cells was 3-105 fold higher than that in benign PNT-2 cells (Figure 1). This result indicated that FABP12 may play an important role in prostate cancer and thus a separate investigation has been conducted to study FABP12. The result on FABP12 study will be published separately from this work.

In this study, the main work was focused on FABP9. At the mRNA level (with a size of 365 KB) [38], its expression in malignant cells was 5-47 fold higher than that in benign cells. When Western blot was used to measure FABP9 protein, its expression was high in highly malignant cell lines PC-3 and PC3-M, whereas its expression in the benign and low-malignant cells was not detectable (Figure 2A). This result demonstrated that elevated levels of FABP9 is correlated with increased malignant characteristics, indicating that FABP9 may play an important role in progression and development of prostate cancer.

The immunohistochemical analysis showed the staining intensity of FABP9 was significantly higher in carcinomas than in BPH (Table 1B) and the increased FABP9 stain was significantly correlated with the increased degree of malignancy, as reflected by the increased GS. Since the combined GS is the most commonly used parameter to stratify the stage of prostate cancer, the fact that the increased FABP9 level is significantly associated with the increased GS suggests that FABP9 expression may be a useful prognostic factor. To confirm this, further assessment was made on the relationship between FABP9 expression and the duration of patient survival time. The result showed that the overall increase in FABP9 was significantly correlated with reduced patient's survival time (log-rank test, *p* < 0.02; Figure 3A). The median survival for patients with weak staining for FABP9 was 60 months which was significantly 2.5- and 3.3-times longer than those of the patients with moderate (24 months) and strong (18 months) stains for FABP9, respectively. The result also showed that patient survival time was significantly associated (log-rank test, *p* < 0.0001; Figure 3D) with increased GS. These findings suggest that increased level of FABP9 expression is significantly correlated with the poor prognosis in terms of patient's survival time, similar to the prognosis made according to GS.

Both PSA and AR index (a parameter used to measure the staining intensity of AR [39]) are used as biomarkers for prostate cancer. Although AR plays a key role in carcinogenesis of prostate cancer, the results in this study showed that the AR index is only of borderline significance in its association with reduced patient survival time (log-rank test, *p* = 0.052; Figure 3B). PSA is the most commonly used biological marker for prostate cancer, but the result in this study showed that correlation between PSA level and patients' survival time was not significantly correlated (log-rank test, *p* = 0.246; Figure 3C). Therefore, neither PSA nor AR index level were reliable prognostic markers for prostate cancer.

When the correlation between FABP9 expression and AR index was assessed, the level of the AR index was significantly higher in cases with strong FABP9 expression than in cases with weak expression of FABP9 (Mann-Whitney *U* test, *p* = 0.03; Figure 4B). In contrast, when FABP9 expression and PSA level was assessed, the differences in patient PSA level were neither significant between strong and weak FABP9 staining (Mann-Whitney *U* test, *p* = 0.142) nor between strong and moderate FABP9 staining (Mann-Whitney *U* test, *p* = 0.098) (Figure 4C). This result showed that there was no correlation between FABP9 levels and PSA levels in blood, and suggested that PSA did not reflect the degree of malignancy of the carcinomas. Like some other previous studies, this work raised questions as to the real value of using PSA as a biomarker for prostate cancer. The real benefit of the widespread use of screening for PSA is still a matter of debate [5, 8, 9]. It is confirmed that only 30% of patients with an abnormal PSA value (above 4 ng/ml) were finally diagnosed with prostate cancer, leading to both an over-biopsy for diagnosis and an over-treatment of low-risk patients[8]. Identification of novel biomarkers that can distinguish between the benign and the malignant nature of each case and can predict the severity of malignancy may provide a new way of disease stratification for prostate cancer and hence reduce unnecessary biopsies (a very invasive procedure) and nonessential treatments. The results in this study suggest that the nature of the patient case and the degree of malignancy are reflected by the level of FABP9 in the primary tumors. The results also suggest that FABP9 is a valuable prognostic marker to predict the outcomes of prostate cancer patients. FABP9 is expressed in the testis and has several important physiological roles in sperm development; including attachment of the acrosome to the sperm nucleus during fertilization and spermatogenesis [20, 40, 41]. Further studies are needed to investigate whether the level of immunoreactive FABP9 can be used to replace (or partially replace) GS so that, at least in some cases, the biopsy procedure can be avoided.

To study the biological significance of increased FABP9 in highly malignant prostate cancer cells, knockdown of FABP9 mRNA was performed via RNAi in highly-expressing PC3-M cells to establish highly- and moderately-suppressed transfectant cell lines, named PC3M-FABP9-siH and PC3M-FABP9-siM, respectively. When tested in an invasion assay, the number of invaded cells from PC3M-FABP9-siH cells, whose level of FABP9 was reduced by 91%, was reduced by 2.8-fold. In contrast that from PC3M-FABP9-siM, whose level of FABP9 was reduced by only 50%, was reduced by 1.6-fold (Figure 5A. d). Thus the reduced level of expression of FABP9 is closely related to the reduced invasive ability of the highly-malignant prostate cancer cells (Figure 5B). This reduction is not achieved by changing the level of FABP5 (Figure 5A. f). This result suggests that FABP9 may play an important role in malignant progression of prostate cancer cells by promoting cellular invasion. More investigations are needed to study the role of FABP9 in carcinogenesis and the possibility of using it as a treatment target. In conclusion, FABP9 is overexpressed in highly malignant prostate cells and tissues. The increased level of FABP9 is significantly correlated with the increased malignancy of the carcinomas, as reflected by the increased GS. FABP9 is also significantly correlated to reduced patient survival time. FABP9 is a more reliable prognostic marker than PSA to predict the outcome of prostate cancer patients and it may play an important role in the invasion of prostate cancer cells.

## MATERIALS AND METHODS

### Cell lines and culture conditions

The following seven cell lines were used: MCF7 breast cancer cell line (the positive control for FABP6), benign prostate epithelial cell line PNT-2 [42, 43], weakly malignant cell line LNCaP derived from moderately-differentiated lymph node metastasis [44], moderately malignant cell line derived from a xenograft 22RV-1 and highly malignant cell lines Du145 derived from brain metastasis [45], PC-3 and PC3-M cell lines derived from poorly-differentiated bone metastasis [46]. All cell lines were grown and maintained as monolayer cultures in RPMI1640 nutrient medium (Invitrogen, Paisly, UK), supplemented with 10% (vol/vol) foetal calf serum, penicillin (100 U/ml) (Biosera, East Sussex, UK), streptomycin (100 μg/ml) and L-glutamine (Invitrogen). For LNCaP cells, sodium pyruvate (100 μg/ml) (Sigma, Grillingham, UK) was also added into the culture medium.

### Quantitative RT-PCR

DNA sequence spanning exon junctions of all *FABP* genes were used to design the real-time PCR primers. The total RNAs were extracted and purified from 6 different cell lines using RNAeasy Mini Kit. The quality and integrity of the mRNA was assessed using RNA 6000 Nano LabChip on Agilent 2100 Bio-analyser. The RNA templates were converted into a complementary DNA (cDNA) using reverse transcriptase. The real-time PCR mixtures for all FABPs and β actin were prepared with 2x brilliant SYBR Green qPCR master mix, 5μM forward primer, 5μM reverse primer, cDNAs and nuclease water. The reactions were centrifuged and placed in a real-time PCR thermocycler. The relative fold differences of FABPs mRNAs between benign and malignant cells were calculated.

### Western blotting

FABP6 and FABP9 expression in prostate cell lines was detected by Western blots using ECL system (Amersham Pharmacia Biotech, Buck, UK) [11]. The polyvinylidene difluoride (PVDF) membrane was first blocked with 5% (v/v) of blocking solution and then incubated with primary antibody, which was either anti-FABP9 antibody (1:500 sheep polyclonal FABP9 antibody, R&D Company) or anti-FABP6 antibody (1:500 Rabbit polyclonal FABP6 antibody, Abcam, UK) for 1 hour at 4°C. The membrane was incubated with secondary antibody conjugated with horseradish peroxidase. The secondary antibody was either rabbit anti-mouse antibody (1:10000) for FABP6 or mouse anti-sheep antibody (1:10000) for FABP9 (Dako, Cambridge, UK). Antibody-bound proteins were visualized by exposure to Kodak XAR-5 film at room temperature. The intensity of the peak areas of the bands was measured using an Alpha Imager 2000 densitometer (Alpha Innotech, Cannock, UK). To correct for possible loading discrepancies, the membrane was incubated with an antibody to β-actin.

### Tissue samples and patient data

Human prostate cancer tissues used in this work were obtained from an archival set of prostate cancer cases that were held in the archives of the original Pathology Department and our Molecular Pathology Laboratory, as described previously [11, 47]. Those patients who were originally diagnosed with prostate cancer but died from other causes were excluded from this study. All samples were taken from patients with an age range of 67 to 73 years who had undergone trans-urethral resection of the prostate (TURP) in the Royal Liverpool University Hospital between 1995 and 2001. This study, approved by the National Science Ethics Committee, was carried out in accordance with the Medical Research Council guidelines (project reference number: Ke; 02/019). All 35 BPH samples and 97 adenocarcinomas had been preserved in 10% (v/v) formalin and embedded in paraffin wax [48]. Cases were re-examined independently by two qualified pathologists and the carcinomas were classified into weakly, moderately and highly malignant tissues according to their combined GS.

### Immunohistochemistry staining

Histological sections were cut from formalin-fixed, paraffin-embedded tissues (4-μm), de-waxed and dehydrated in xylene and ethanol, respectively [47]. Tissues were incubated in methanol and hydrogen peroxide (3%) for 12 min. Sections were incubated with primary anti-FABP9 antibody (1:500 sheep polyclonal FABP9 antibody, R&D Company) or with anti-FAB6 antibody (1:50 rabbit polyclonal FABP6 antibody, Sigma-Aldrich, UK) at room temperature for 1 hour and then incubated with a rabbit anti-sheep IgG linker or mouse anti-rabbit IgG linker (Vector Laboratories, Burlingame, CA, USA) for 30 min. Sections were incubated with Envision™ FLEX DAB+ chromogen mixed with Envision™ FLEX substrate (ldop/ml) (Dakocytomation, Ely, UK) for 15min. All sections were counterstained with hematoxylin, mounted on glass slides with cover slip using DPX mountant, and observed by light microscope for scoring.

### Scoring immunoreactivity

A standard light microscope (x400) was used to evaluate cytoplasmic and nuclear expression of FABP6 and FABP9. Immunoreactivity was examined independently by two qualified observers. The intensity of cytoplasmic staining was classified into 4 categories: unstained, weakly, moderately and strongly stained which were expressed as 0 (−), 1 (+), 2 (++) and 3 (+++), respectively. Nuclear staining was assessed by the intensity of staining which was expressed as 0 (−), 1 (+), 2 (++) and 3 (+++). The differences in scoring categories between 2 observers were <5%.

### Statistical analysis

The analysis was performed using the Statistical Package for Social Sciences (SPSS). Correlation between FABP6 and FABP9 expression and the benign and malignant prostate tissues were assessed by χ^2^ test. Kaplan Meier plots were used to separate survival times of patients with differentially stained samples for FABP6 or FABP9. The graphs were labelled the significance association with Log Rank test (*p* value). Correlation between FABP9 and GS, AR or PSA level was assessed via box plot and Mann Whitney *U* 2x3 tests. Statistical significance was defined as *P* < 0.05.

### RNA interference

Bioinformatics & Research computing Software (Whitehead siRNA selection program) was used to select three different candidate sequences for FABP9 siRNA. Specificity of these sequences to FABP9 was confirmed by Blast search. Oligonucleotides made from these 3 sequences were commercially purchased (Ambion Life technologies, USA):

Sequence 1, sense strand 5′: GTACCTCGGTTAGTGAAACCGACAGTTCAAGAG; antisense strand 5′: AGCTTTTCCAAAAAGGTTAGTGAAACCGACAGTCTCTTGAACTGTCGGTTTCACTAACCGAG. Sequence 2, sense strand 5′: GTACCTCCTCAATGATTCACGTCCAATCAAGAGTTGGACGTGAATCATTGAGTTTTTGGAAA; antisense strand 5′: AGCTTTTCCAAAAACTCAATGATTCACGTCCAACTCTTGATTGGACGTGAATCATG. Scramble negative control, sense 5′: GTACCTCGCAGACCTTCCCATATAATTCAAGAGATTATATGGGAAGGTCTGCTTTTTGGAAA; antisense 5′: AGCTTTTCCAAAAAGCAGACCTTCCCATATAATCTCTTGAATTATATGGGAAGGTCTGCGAG.

PC3-M cell line was transfected transiently with the X-tremeGENE siRNA Transfection Reagent (Roche) and the level of FABP9 was measured by Western blot analysis after the transient transfection of each of the 3 candidate sequences. The most efficient suppressor sequence was identified from the blot and was used to design short hairpin RNA (shRNA) using siRNA Wizard™ Software(InvivoGen, USA). Two shRNA inserts containing RNAi sequences against FABP9 were cloned into the psiRNA-h7SKGFPzeo plasmid (InvivoGen, USA) separately. PC3-M was stably transfected with vectors containing different FABP9 shRNAs using X-tremeGENE HP DNA Transfection Reagent (Roche, Germany). In a separate transfection, a vector containing scrambled RNA was used as a control. Transfected cells were cultured in a selective medium containing Zeocin (100μg/ml) (Life Technologies) for 3-4 weeks until the colonies were visualized. Ring cloning technique was used to isolate five single colonies to establish FABP9-supressed PC3-M transfectants and the level of FABP9 in these transfectants was measured by Western blot analysis.

### Invasion assay

The invasiveness of the transfectants was assessed in a BD BioCoat™ Matrigel™ Invasion Chamber (BD Biosciences, USA). After 24-hour starvation, PC3-M and transfected cells at a density of 2.5x10^4^ cells/ml were loaded into every upper compartment of chambers. Routine medium was loaded in the lower compartment and serum-free medium was used as negative control for each cell line. The chambers were maintained in a humidified tissue culture incubator at 37°C, 5% CO_2_ (v/v). After 24-hour cells in the upper compartment were removed and washed with PBS. Then, the cells, which invaded the lower part, were fixed and stained with 2% (v/v) crystal violet for 10 minutes. The number of invaded cells was counted using a light microscope.

## References

[1] De Angelis R, Sant M, Coleman MP, Francisci S, Baili P, Pierannunzio D, Trama A, Visser O, Brenner H, Ardanaz E, Bielska-Lasota M, Engholm G, Nennecke A, Siesling S, Berrino F, Capocaccia R (2014). Cancer survival in Europe 1999-2007 by country and age: results of EUROCARE--5-a population-based study. Lancet Oncol.

[2] Torre LA, Bray F, Siegel RL, Ferlay J, Lortet-Tieulent J, Jemal A (2015). Global cancer statistics, 2012. CA Cancer J Clin.

[3] Brawley OW (2012). Trends in prostate cancer in the United States. J Natl Cancer Inst Monogr.

[4] Barry MJ (2001). Prostate-Specific–Antigen Testing for Early Diagnosis of Prostate Cancer. New England Journal of Medicine.

[5] Moore AL, Dimitropoulou P, Lane A, Powell PH, Greenberg DC, Brown CH, Donovan JL, Hamdy FC, Martin RM, Neal DE (2009). Population-based prostate-specific antigen testing in the UK leads to a stage migration of prostate cancer. BJU Int.

[6] Adamson J, Morgan EA, Beesley C, Mei Y, Foster CS, Fujii H, Rudland PS, Smith PH, Ke Y (2003). High-level expression of cutaneous fatty acid-binding protein in prostatic carcinomas and its effect on tumorigenicity. Oncogene.

[7] Sharifi N, Gulley JL, Dahut WL (2005). ANdrogen deprivation therapy for prostate cancer. JAMA.

[8] Schröder FH, Hugosson J, Roobol MJ, Tammela TLJ, Ciatto S, Nelen V, Kwiatkowski M, Lujan M, Lilja H, Zappa M, Denis LJ, Recker F, Berenguer A, Määttänen L, Bangma CH, Aus G (2009). Screening and Prostate-Cancer Mortality in a Randomized European Study. New England Journal of Medicine.

[9] Andriole GL, Crawford ED, Grubb RLI, Buys SS, Chia D, Church TR, Fouad MN, Gelmann EP, Kvale PA, Reding DJ, Weissfeld JL, Yokochi LA, O'Brien B, Clapp JD, Rathmell JM, Riley TL (2009). Mortality Results from a Randomized Prostate-Cancer Screening Trial. New England Journal of Medicine.

[10] Jing C, Beesley C, Foster CS, Chen H, Rudland PS, West DC, Fujii H, Smith PH, Ke Y (2001). Human cutaneous fatty acid-binding protein induces metastasis by up-regulating the expression of vascular endothelial growth factor gene in rat Rama 37 model cells. Cancer Res.

[11] Forootan SS, Wong YC, Dodson A, Wang X, Lin K, Smith PH, Foster CS, Ke Y (2007). Increased Id-1 expression is significantly associated with poor survival of patients with prostate cancer. Hum Pathol.

[12] Sardana G, Diamandis EP (2012). Biomarkers for the diagnosis of new and recurrent prostate cancer. Biomark Med.

[13] Bhavsar T, McCue P, Birbe R (2013). Molecular diagnosis of prostate cancer: are we up to age?. Semin Oncol.

[14] Jing C, Beesley C, Foster CS, Rudland PS, Fujii H, Ono T, Chen H, Smith PH, Ke Y (2000). Identification of the messenger RNA for human cutaneous fatty acid-binding protein as a metastasis inducer. Cancer Res.

[15] Bao Z, Malki MI, Forootan SS, Adamson J, Forootan FS, Chen D, Foster CS, Rudland PS, Ke Y (2013). A novel cutaneous Fatty Acid-binding protein-related signaling pathway leading to malignant progression in prostate cancer cells. Genes Cancer.

[16] Morgan EA, Forootan SS, Adamson J, Foster CS, Fujii H, Igarashi M, Beesley C, Smith PH, Ke Y (2008). Expression of cutaneous fatty acid-binding protein (C-FABP) in prostate cancer: potential prognostic marker and target for tumourigenicity-suppression. Int J Oncol.

[17] Forootan FS, Forootan SS, Malki MI, Chen D, Li G, Lin K, Rudland PS, Foster CS, Ke Y (2014). The expression of C-FABP and PPARgamma and their prognostic significance in prostate cancer. Int J Oncol.

[18] Forootan FS, Forootan SS, Gou X, Yang J, Liu B, Chen D, Al Fayi MS, Al-Jameel W, Rudland PS, Hussain SA, Ke Y (2016). Fatty acid activated PPARgamma promotes tumorigenicity of prostate cancer cells by up regulating VEGF via PPAR responsive elements of the promoter. Oncotarget.

[19] Furuhashi M, Hotamisligil GS (2008). Fatty acid-binding proteins: role in metabolic diseases and potential as drug targets. Nat Rev Drug Discov.

[20] Smathers RL, Petersen DR (2011). The human fatty acid-binding protein family: evolutionary divergences and functions. Hum Genomics.

[21] Chmurzynska A (2006). The multigene family of fatty acid-binding proteins (FABPs): function, structure and polymorphism. J Appl Genet.

[22] Schachtrup C, Emmler T, Bleck B, Sandqvist A, Spener F (2004). Functional analysis of peroxisome-proliferator-responsive element motifs in genes of fatty acid-binding proteins. Biochem J.

[23] Gao N, Qu X, Yan J, Huang Q, Yuan HY, Ouyang DS (2010). L-FABP T94A decreased fatty acid uptake and altered hepatic triglyceride and cholesterol accumulation in Chang liver cells stably transfected with L-FABP. Mol Cell Biochem.

[24] Pinthus JH, Lu JP, Bidaisee LA, Lin H, Bryskine I, Gupta RS, Singh G (2007). Androgen-dependent regulation of medium and long chain fatty acids uptake in prostate cancer. Prostate.

[25] Hammamieh R, Chakraborty N, Barmada M, Das R, Jett M (2005). Expression patterns of fatty acid binding proteins in breast cancer cells. J Exp Ther Oncol.

[26] Boiteux G, Lascombe I, Roche E, Plissonnier ML, Clairotte A, Bittard H, Fauconnet S (2009). A-FABP, a candidate progression marker of human transitional cell carcinoma of the bladder, is differentially regulated by PPAR in urothelial cancer cells. Int J Cancer.

[27] Ohmachi T, Inoue H, Mimori K, Tanaka F, Sasaki A, Kanda T, Fujii H, Yanaga K, Mori M (2006). Fatty acid binding protein 6 is overexpressed in colorectal cancer. Clin Cancer Res.

[28] Sinha P, Hutter G, Kottgen E, Dietel M, Schadendorf D, Lage H (1999). Increased expression of epidermal fatty acid binding protein, cofilin, and 14-3-3-sigma (stratifin) detected by two-dimensional gel electrophoresis, mass spectrometry and microsequencing of drug-resistant human adenocarcinoma of the pancreas. Electrophoresis.

[29] Kawaguchi K, Senga S, Kubota C, Kawamura Y, Ke Y, Fujii H (2016). High expression of Fatty Acid-Binding Protein 5 promotes cell growth and metastatic potential of colorectal cancer cells. FEBS Open Bio.

[30] Li H, Lu Q, Dong LH, Xue H, Zhou HY, Yang HJ (2007). [Expression of fatty acid binding protein in human breast cancer tissues]. Xi Bao Yu Fen Zi Mian Yi Xue Za Zhi.

[31] Teratani T, Domoto T, Kuriki K, Kageyama T, Takayama T, Ishikawa A, Ozono S, Nozawa R (2007). Detection of transcript for brain-type fatty Acid-binding protein in tumor and urine of patients with renal cell carcinoma. Urology.

[32] Slipicevic A, Jorgensen K, Skrede M, Rosnes AK, Troen G, Davidson B, Florenes VA (2008). The fatty acid binding protein 7 (FABP7) is involved in proliferation and invasion of melanoma cells. BMC Cancer.

[33] Sharma MK, Liu RZ, Thisse C, Thisse B, Denovan-Wright EM, Wright JM (2006). Hierarchical subfunctionalization of fabp1a, fabp1b and fabp10 tissue-specific expression may account for retention of these duplicated genes in the zebrafish (Danio rerio) genome. FEBS J.

[34] Venkatachalam AB, Thisse C, Thisse B, Wright JM (2009). Differential tissue-specific distribution of transcripts for the duplicated fatty acid-binding protein 10 (fabp10) genes in embryos, larvae and adult zebrafish (Danio rerio). FEBS J.

[35] Agulleiro MJ, Andre M, Morais S, Cerda J, Babin PJ (2007). High transcript level of fatty acid-binding protein 11 but not of very low-density lipoprotein receptor is correlated to ovarian follicle atresia in a teleost fish (Solea senegalensis). Biol Reprod.

[36] Hotamisligil GS, Johnson RS, Distel RJ, Ellis R, Papaioannou VE, Spiegelman BM (1996). Uncoupling of obesity from insulin resistance through a targeted mutation in aP2, the adipocyte fatty acid binding protein. Science.

[37] Uehara H, Takahashi T, Oha M, Ogawa H, Izumi K (2014). Exogenous fatty acid binding protein 4 promotes human prostate cancer cell progression. Int J Cancer.

[38] Stejskal D, Karpisek M, Bronsky J (2008). Serum adipocyte-fatty acid binding protein discriminates patients with permanent and temporary body weight loss. J Clin Lab Anal.

[39] Pertschuk LP, Schaeffer H, Feldman JG, Macchia RJ, Kim YD, Eisenberg K, Braithwaite LV, Axiotis CA, Prins G, Green GL (1995). Immunostaining for prostate cancer androgen receptor in paraffin identifies a subset of men with a poor prognosis. Lab Invest.

[40] Oko R, Morales CR (1994). A novel testicular protein, with sequence similarities to a family of lipid binding proteins, is a major component of the rat sperm perinuclear theca. Dev Biol.

[41] Kido T, Namiki H (2000). Expression of testicular fatty acid-binding protein PERF 15 during germ cell apoptosis. Dev Growth Differ.

[42] Berthon P, Cussenot O, Hopwood L, Leduc A, Maitland N (1995). Functional expression of sv40 in normal human prostatic epithelial and fibroblastic cells - differentiation pattern of nontumorigenic cell-lines. Int J Oncol.

[43] Cussenot O, Berthon P, Berger R, Mowszowicz I, Faille A, Hojman F, Teillac P, Le Duc A, Calvo F (1991). Immortalization of human adult normal prostatic epithelial cells by liposomes containing large T-SV40 gene. J Urol.

[44] Horoszewicz JS, Leong SS, Kawinski E, Karr JP, Rosenthal H, Chu TM, Mirand EA, Murphy GP (1983). LNCaP model of human prostatic carcinoma. Cancer Res.

[45] Stone KR, Mickey DD, Wunderli H, Mickey GH, Paulson DF (1978). Isolation of a human prostate carcinoma cell line (DU 145). Int J Cancer.

[46] Kaighn ME, Lechner JF, Narayan KS, Jones LW (1978). Prostate carcinoma: tissue culture cell lines. Natl Cancer Inst Monogr.

[47] Forootan SS, Foster CS, Aachi VR, Adamson J, Smith PH, Lin K, Ke Y (2006). Prognostic significance of osteopontin expression in human prostate cancer. Int J Cancer.

[48] Foster CS, Gosden CM, Ke YQ (2006). Primer: tissue fixation and preservation for optimal molecular analysis of urologic tissues. Nat Clin Pract Urol.

